# Subcutaneous rupture of the Achilles tendon and ipsilateral fracture of the medial malleolus

**DOI:** 10.1186/1471-2474-7-59

**Published:** 2006-07-27

**Authors:** Nicola Maffulli, Paula J Richards

**Affiliations:** 1 Department of Trauma and Orthopaedic Surgery, Keele University School of Medicine, Stoke-on-Trent, UK; 2 Department of Imaging, University Hospital of North Staffordshire, Stoke-on-Trent, UK

## Abstract

**Background:**

Although ankle fractures and an Achilles tendon rupture are relatively frequent in isolation, their association in the same injury is uncommon.

**Case presentation:**

A 38 year old male tree surgeon fell six meters from a tree, sustaining a subcutaneous rupture of the Achilles tendon and an ipsilateral closed fracture of the medial malleolus. The injuries were diagnosed following clinical examination and imaging.

**Conclusion:**

This injury combination is infrequent, and management of the Achilles tendon rupture should take into account the necessity not to secondarily displace the fracture of the medial malleollus.

## Background

Although ankle fractures and an Achilles tendon rupture are relatively frequent in isolation [1,2], their association in the same injury is uncommon [3-7]. The tendon of the long flexors [8] can be trapped the fracture site and thus rupture. These injuries are usually diagnosed at the time of open reduction and internal fixation of the ankle fracture. A different mechanism of injury may cause the association between the Achilles tendon rupture and an ankle fracture, and this injury can be initially undiagnosed [4-7].

## Case presentation

**Figure 1:**
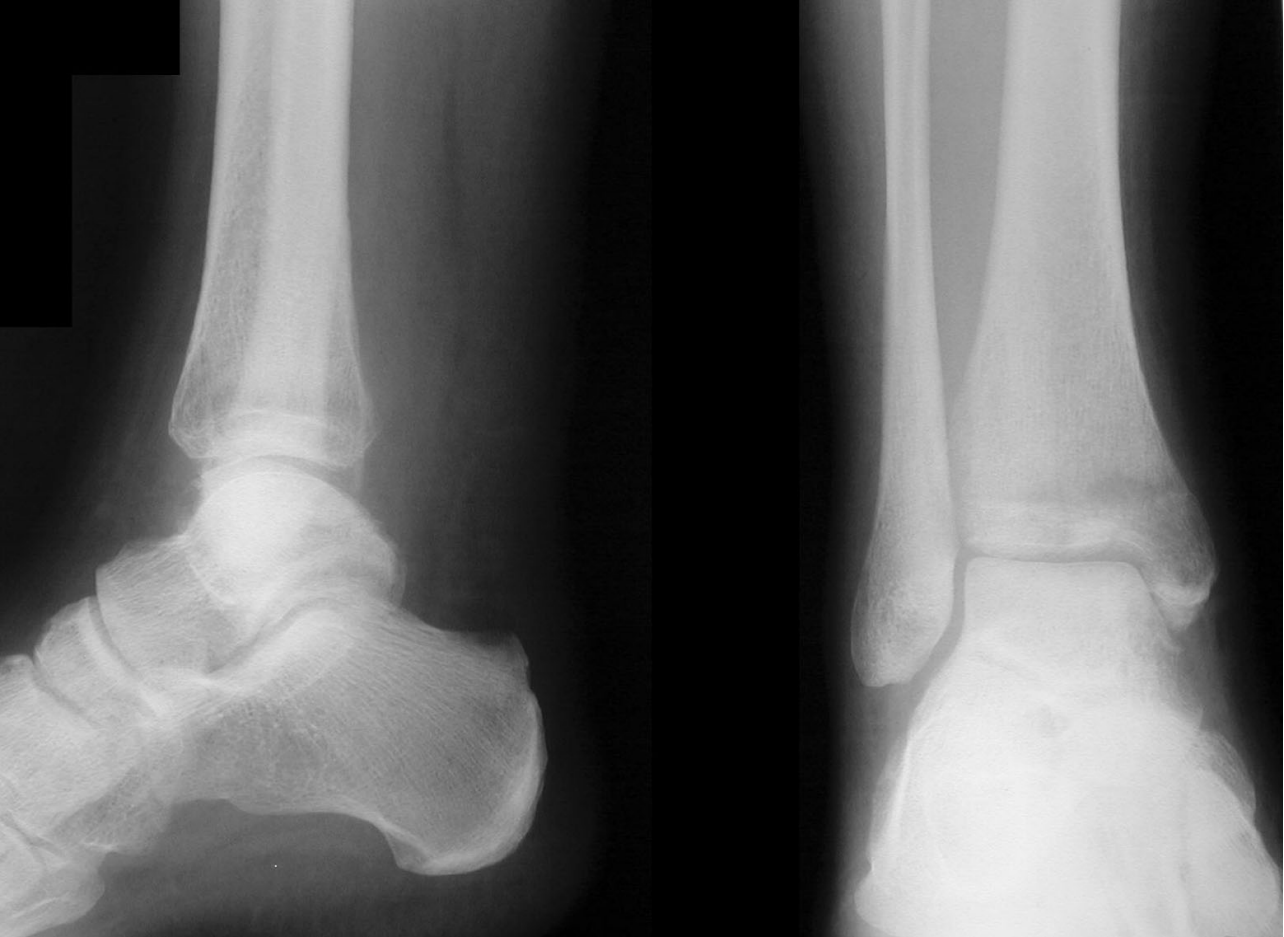
Radiograph of the ankle at presentation: an undisplaced fracture of the medial malleolus is evident.

**Figure 2:**
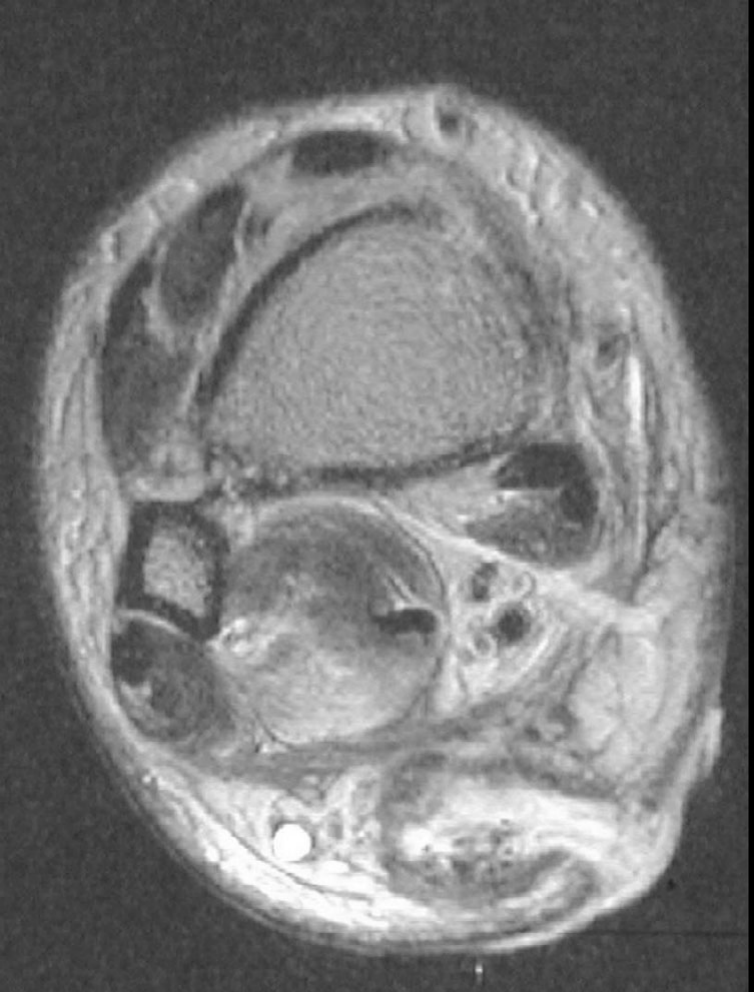
MRI scan at presentation, cross sectional view at the level of the upper end of the distal stump of the Achilles tendon. Note the disruption of the medial cortex of the tibia, indicating the presence of a fracture in that area.

A 38 year old male tree surgeon fell six meters from a tree. He was wearing reinforced work boots, and, during the fall, he struck one tree branch with the plantar aspect of his right forefoot before landing on the ground and falling on his back. At examination in the local Accident and Emergency Department, he referred pain and tenderness over the posterior aspect of the ankle, and swelling was noted posteromedially. At palpation, there was tenderness four centimetres above the insertion of the Achilles tendon on the calcaneal tuberosity, a two centimetre gap in the Achilles tendon was present, and the calf squeeze test was positive for a subcutaneous Achilles tendon rupture [9]. Plain radiographs revealed an undisplaced fracture of the medial malleolus (Fig 1). Both lesions wee further confirmed by a magnetic resonance scan performed for scientific documentation (Fig 2).

The patient initially elected to be managed conservatively, and received an above knee plaster of Paris cast with the foot in maximal equines. He was kept non-weight bearing for one week, and was referred to our unit, where he was seen after two weeks following the original injury. At that time, the above clinical findings were confirmed, and the patient requested to undergo surgery.

At surgery, the patient was placed prone on a fracture table in a bloodless field furnished by a thigh tourniquet. The medial malleolar fracture was visualised under image intensifier: it was undisplaced, and it was decided to manage it conservatively if still undisplaced by the end of the Achilles tendon repair. The tendon was exposed using a slightly curvilinear medial approach. An end-to-end repair was performed using a single modified Kessler suture with No 1 Vicryl (Polyglactin 910 braided absorbable suture, Johnson & Johnson, European Logistics Centre, 66 Rue de la Fusee, B-1130 Bruxelles, Belgium). A running circumferential suture with 3-0 Vicryl reinforced the core suture. The repair was thicker than the original tendon, and the paratenon could not be sutured over it. Continuous 4-0 Vicryl reabsorbable sutures were used for the subcutaneous fat, and the skin was closed with subcuticular 4-0 Vicryl reabsorbable suture. At the end of the procedure, the fracture of the medial malleolus was undisplaced, and it was decided to leave it alone. The skin wound was dressed with gauze, sterile plaster wool was applied, followed by a below knee synthetic cast without increasing the natural minimal equinus of the ankle.

When the cast had dried, the patient was encouraged to mobilise with the use of crutches, under the direction of a physiotherapist. The patient was discharged the day after the operation. He was allowed to bear weight on the tip toes of the operated leg as tolerated, but was told to keep the leg elevated as much as possible for the first two post-operative weeks. Two weeks after the operation, a synthetic anterior below knee slab was applied, with the ankle in the natural minimal equinus. The synthetic slab was secured to the leg with three or four removable Velcro (Velcro USA Inc., Manchester, NH, USA) straps for four weeks. The patient was encouraged to weight bear on the operated limb as soon as he felt comfortable, and to gradually progress to full weight bearing. The patient was seen by a trained physiotherapist who taught him gentle mobilisation exercises of the ankle, isometric contraction of the gastrosoleus complex, and gentle concentric contraction of the calf muscles. The patient was encouraged to perform mobilisation of the involved ankle several times per day after unstrapping the two most distal Velcro straps. The patient was given an appointment six weeks from the operation, when the anterior slab was removed [10].

At that time, the fracture site was not painful, the patient was able to fully weight bear and started more intensive physiotherapy. At the 12 month follow up, the patient was asymptomatic with a full range of active and passive ankle motion. The patient was able to walk on tip toes unaided, and had returned to his occupation as a tree surgeon. He reported that, following a full day at work, his ankle at times swelled up, though the swelling had resolved by the following morning. He was discharged from further follow up, and, on telephone questioning two years following the injury, he reported to be leading a normal life.

## Conclusion

To our knowledge, only five single case reports describe an ankle fracture combined with an Achilles tendon rupture [3-7]. Each patient sustained a complete rupture of the Achilles tendon. The medial malleolus fracture was a closed oblique or vertical fracture, and the lateral malleolus was intact. The injury mechanism consists of a sudden force applied to the forefoot with ankle hyperextension [6,7] or hind-foot inversion [4,5]. Our patient impacted his forefoot on at least one tree branch falling down, and is likely to have experienced sudden ankle hyperextension, a mechanism which, coupled with contraction of the triceps surae muscle, is likely to have produced the Achilles tendon rupture [11]. Ankle hyperextension can by itself produce ankle fractures [12].

Either the Achilles tendon rupture or the fracture of the medial malleolus can be missed at initial examination, but this combination of injuries may be more frequent than we report: in Achilles tendon ruptures resulting from skiing injuries, a non-displaced medial malleolus fracture occasionally occurs, but did not give data [13]. Also, Lugger et al. [14], reporting on alpine skiing injuries collected mainly in the 1960's, found a 5.3% incidence of medial malleolar fractures alpine skiers who sustained an Achilles tendon rupture [14]. Modern high, rigid moulded ski boots limit greatly ankle motion, and ski bindings with automatic posterior release prevent excessive posterior stretching.

Forceful overload of the forefoot may well have preceded the malleolar fracture, and produced the Achilles tendon rupture, instead of a syndesmosis injury or high fibular fracture. Assal et al [3] recommend that patients presenting with a supination-adduction ankle injury (Weber Type A fracture) should have a thorough examination of the Achilles tendon. Additionally, patients presenting with a traumatic Achilles tendon rupture should have routine antero-posterior and lateral radiographs of the ankle to rule out an associated bony lesion. Good clinical practice would dictate that thorough systematic examination should be continued even though a lesion has already been identified [3,6]. We agree with this suggestion.

## Competing interests

The author(s) declare that they have no competing interests.

## Authors' contributions

NM was referred the patient, made the correct diagnosis, formulated the management plan, carried out the clinical management, and wrote the first draft of the manuscript.

PJR performed the imaging studies, interpreted them, participated in the design of the study, and reviewed the manuscript. All authors read and approved the final manuscript.

## Pre-publication history

The pre-publication history for this paper can be accessed here:



## Figures and Tables

**Figure 3:**
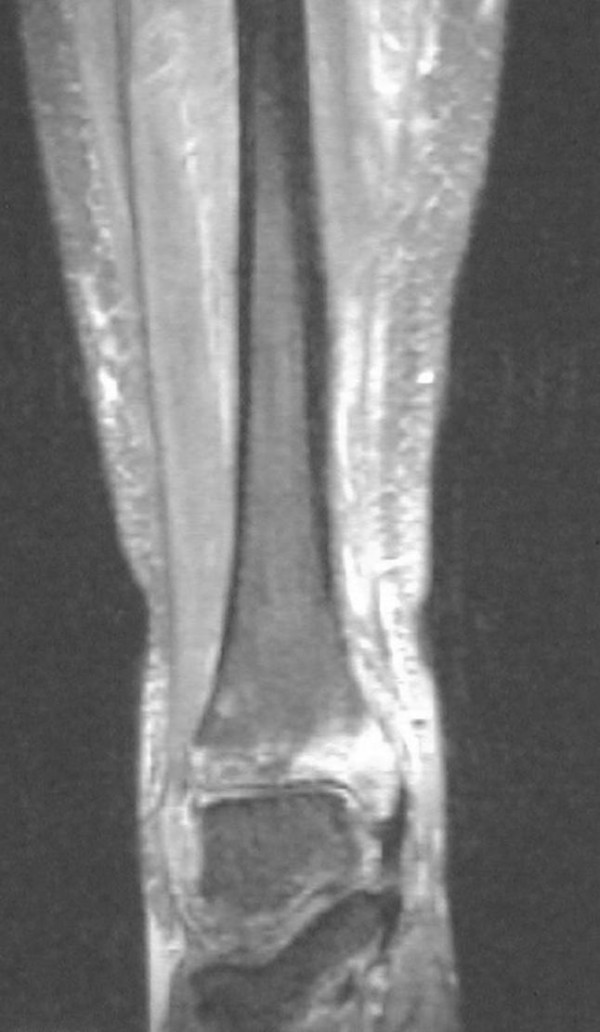
MRI scan one week after open repair of the Achilles tendon. Note the fracture of the medial malleolus

**Figure 4:**
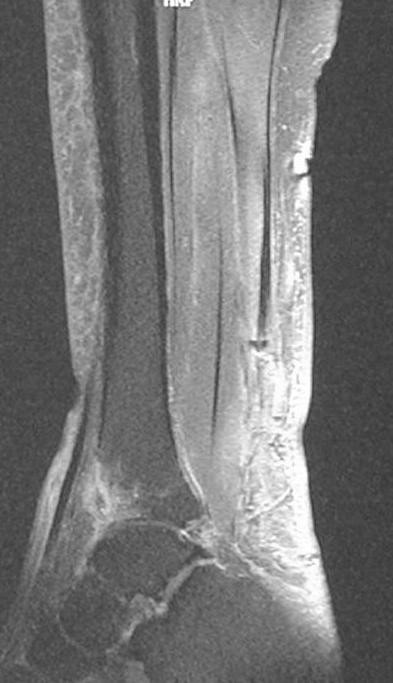
MRI scan one week after open repair of the Achilles tendon. Note the dishomogeneous signal from the repaired Achilles tendon and the bone bruise

**Figure 5:**
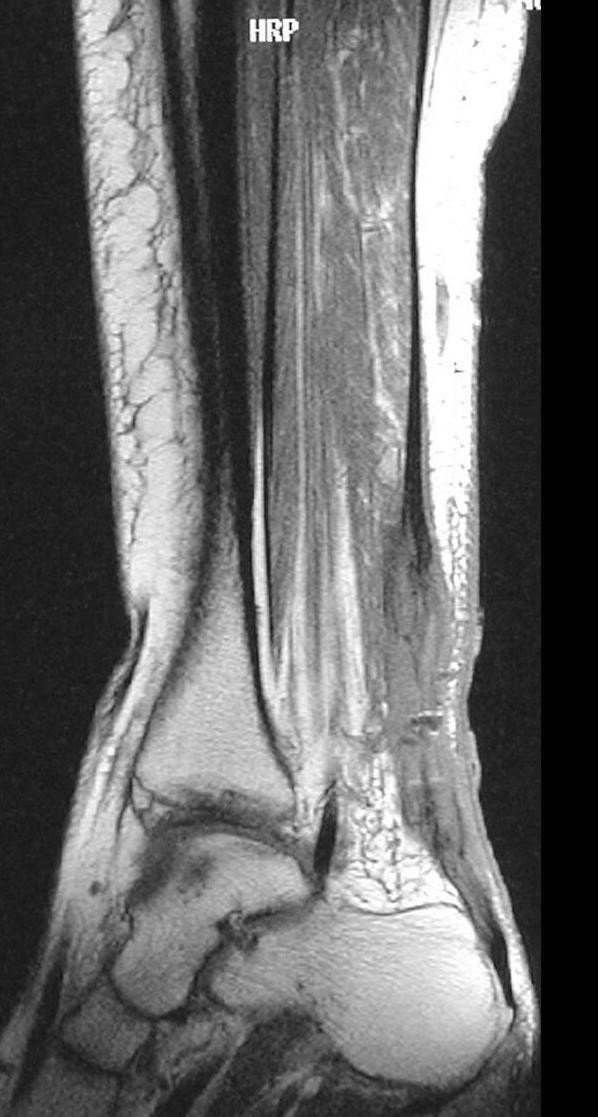
MRI scan one week after open repair of the Achilles tendon. Note the fracture line of the medial malleolus.
